# Epstein-Barr Virus-Associated T-Cell Lymphoproliferative Disorder Presenting as Chronic Diarrhea and Intestinal Bleeding: A Case Report

**DOI:** 10.3389/fimmu.2018.02583

**Published:** 2018-11-16

**Authors:** Yaxin Wang, Yajun Li, Xiangwei Meng, Xiumei Duan, Meilan Wang, Wenqing Chen, Tongyu Tang, Yuqin Li

**Affiliations:** ^1^Department of Gastroenterology, Ordos Central Hospital, Dongsheng, China; ^2^Department of Gastroenterology, Bethune First Affiliated Hospital of Jilin University, Changchun, China; ^3^Department of Pathology, Bethune First Affiliated Hospital of Jilin University, Changchun, China; ^4^Department of Gastroenterology, The Hospital of Jilin Province, Changchun, China

**Keywords:** Epstein-Barr virus infection, T-cell lymphoproliferative disease, clinical features, endoscopic features, differential diagnosis

## Abstract

Systemic Epstein-Barr virus-positive T-cell lymphoproliferative childhood disease (EBV+ T-LPD) is extremely rare. Primary acute or chronic active Epstein-Barr virus infection triggers EBV+ T-LPD's onset and the disease involves clonal proliferation of infected T-cells with activated cytotoxic phenotype. The adult-onset EBV+ T-LPD (ASEBV+ T-LPD) is even rarer and needs to be extensively studied. Further, according to literature review, it is a challenge to find patients who are immunocompetent and diagnosed with ASEBV+ T-LPD involving gastrointestinal tract. This case report discusses a previously healthy middle aged woman who presented with unique symptoms mimicking inflammatory bowel disease, and required a total colectomy and terminal ileum rectomy, as reveled by endoscopic examinations, due to severe gastrointestinal bleeding. Post-surgery histopathological findings were confirmatory for the diagnosis of ASEBV+ T-LPD (II: Borderline). This patient died 7 months after the diagnosis.

## Introduction

Epstein-Barr virus (EBV), a ubiquitous DNA virus, belongs to the γ subfamily of herpesviruses. It is estimated that almost 95% of adults are infected with EBV. A majority of EBV infections take place during early childhood life and are mostly asymptomatic. However, infections in adolescents or young adults are reported to result in infectious mononucleosis (IM) with patients often presenting with fever, pharyngitis, lymphadenopathy, and splenomegaly (1, 2). Onset of IM involves a transient proliferation of EBV-infected B cells, in addition to an excessive response of cytotoxic T cells (CTL) that are EBV-specific (3). In a majority of patients, these IM-symptoms resolve without sequelae. However, in rare patients, even without apparent immunodeficiency, the disease manifests into chronic EBV infection, which is marked by persistent IM-like symptoms, termed “chronic active EBV disease (CAEBV).” This condition is included in the 2008 World Health Organization (WHO) classification of lymphomas as the systemic EBV+ T-cell LPD of childhood. Patients have a characteristically high load of EBV-DNA in their peripheral blood and a systemic clonal expansion of EBV-infected T cells or the natural killer (NK) cells (3, 4).

The reports on ASEBV+T-LPD are sporadic and rare (5) and gastro-intestinal symptoms of ASEBV(+)T-LPD are even rarer. These symptoms are often misdiagnosed as tuberculosis and inflammatory bowel diseases. Here, we report the case of an immunocompetent adult patient who presented with a primary complaint of chronic diarrhea, abdominal pain and slight hematochezia, but was finally diagnosed with EBV-associated T-cell LPD (II: Borderline). The aim of this report and our analysis is to promote the recognition of intestinal lesions of the ASEBV+ T-LPD.

## Consent

The patient consented to participate in the study and provided a written consent to publish this case report.

## Case presentation

A previously healthy 43-years-old woman living in pasturing area, with no personal or family history of immunodeficiency, presented with a 2-months history of intermittent fever that was sometimes accompanied with chill, abdominal pain, diarrhea and hematochezia.

### Initial presentation

The woman reported to a local hospital initially, where she was diagnosed with inflammatory bowel disease and treated with clindamycin, resulting in some clinical improvement. When her previous symptoms deteriorated for 10 days, she was seen at the Gastroenterology Department at our hospital. An X-ray of patient's abdomen at out-patient department showed signs of “incomplete intestinal obstruction” and she was admitted for further evaluation. Her physical examination was unremarkable, except for low blood pressure (97/71 mmHg) and a pale appearance. There was no self-reported loss of weight/appetite or other significant clinical findings at initial presentation. The laboratory tests at this initial presentation are summarized in Table 1.

**Table 1:** Laboratory results of patient at initial presentation.

**Laboratory items**	**Laboratory results**	
Hemoglobin	82 g/L	(113–151) g/L
Neutrophile percentage	0.77	(50–70) %
Albumin	30.2 g/L	(35–52) g/L
C-reactive protein	123.0 mg/L	(0–5) mg/L
Erythrocyte sedimentation rate	42 mm/1 h	(0-20) mm/1 h
D-Dimer level	273 μg/L	(< 232) μg/L

### Treatment

**Figure 1:**
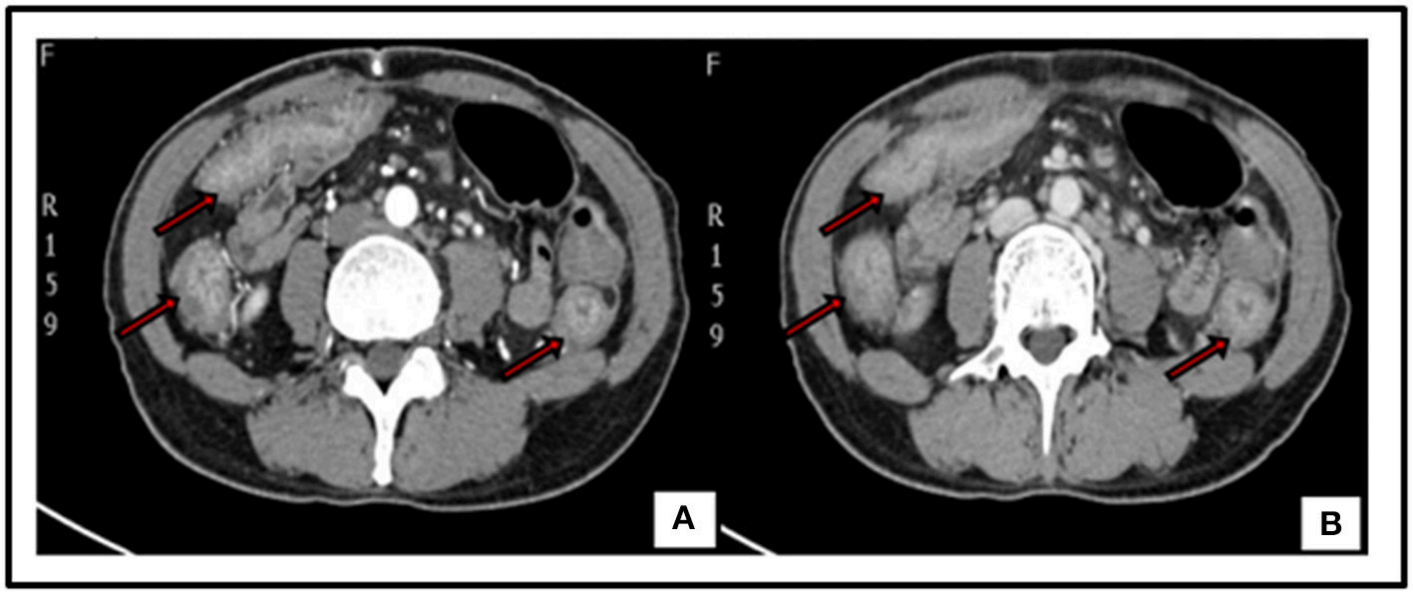
Enhanced-CT of abdomen: diffusible thickening mucosa of ascending, transverse and descending colon with coarse rims, uneven enhancement in artery phase (Red arrow) **(A)**. Image of Venous phase (Red arrow) **(B)**. Multiple lymph nodes could be seen in mesenteric and posterior-peritoneum areas.

**Figure 2:**
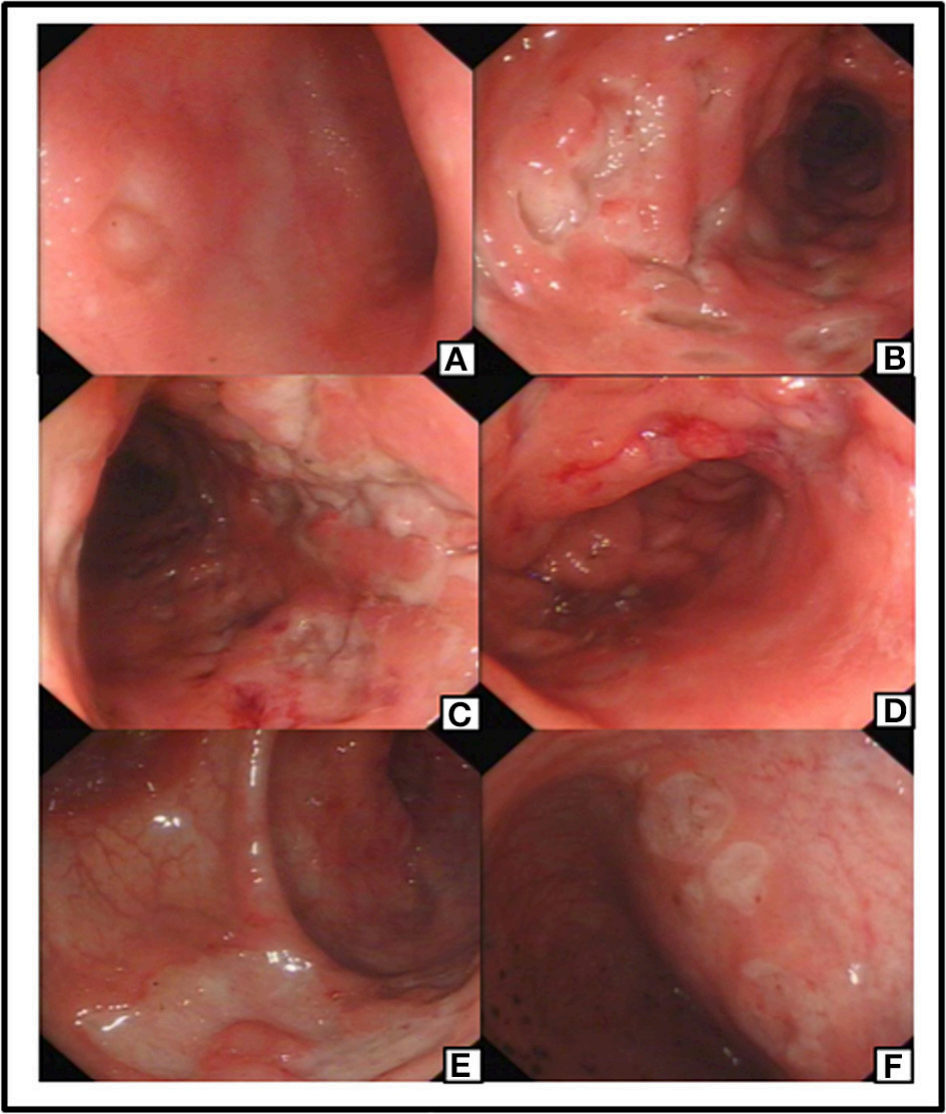
Preoperative colonoscopy manifestation: an ulceration with clear rim and base revealed in ileum terminal **(A)**. Preoperative colonoscopy revealed multiple and discrete ulcers as visible in different segment of Colon **(B–E)** and Rectum **(F)**.

The patient was treated with anti-infective and symptomatic therapy initially. An enhanced-CT scan performed on day 2 in the hospital showed diffusible change in ascending, transverse and descending colon mimic ulcerous colitis. Multiple lymph nodes of mesenteric and posterior-peritoneum areas were visible (Figure 1). An emergency colonoscopy examination was suggested, which revealed multiple, discrete ulcers with irregular boundaries and clean base, scattered throughout the colon. The diameters of ulcers varied from 6 to 30 mm and errhysis could be seen around the erosion. Normal mucosa was also clearly visible amid the ulcers (Figure 2).

The specific clonoscopic presentation made a strong indication of inflammatory disease including Ulcerative colitis (UC) and Crohn Disease (CD). Specific infectious bowel disease, especially intestinal tuberculosis, was also suspected because of patient's persistent fever, and the intestinal lymphoma diagnosis also could not be excluded. Further laboratory tests were done, including, chronic inflammatory enteropathy combination, anti-nuclear antibody (ANA), anti-neutrophil cytoplasmic antibodies (ANCA), rheumatism related factors (ASO+RF+ CRP), tumor markers of digestive tract, procalcitonin (PCT), blood culture, stool culture, amebic trophozoite detection, cytomegalovirus (CMV), anti-EBV antibodies, T-cell spot experiment and PPD test for *Mycobacterium tuberculosis*, Widal reaction, anti-Brucella antibodies, hepatitis virus indicators, Fungi D glucan detection, etc. Results for all of these tests were either negative or unremarkable, except for the high EBV-related antibody titers: anti-EBV viral capsid antigen (VCA)-IgG: (4.157 s/co), anti-EBV VCA-IgM (0.391 s/co), Epstein barr nuclear antigen (EBNA) IgG (0.865 s/co), EBEA IgG (1.933 s/co), and Epstein barr early antigen (EBEA) IgM (0.187 s/co).

Patient showed no signs of remission during hospitalization. On day 10, a color Doppler ultrasound of abdomen showed splenomegaly. Patient developed severe intestinal bleeding 14 days after admission and underwent an emergency total colectomy, terminal ileum ectomy, small intestine and rectum anastomosis and preventive ileostomy. She was discharged after stable condition and was treated with mesalazine for ulcerative colitis (UC), based on the post-surgery pathological diagnosis made by our hospital. Mesalazine was stopped after a few courses.

**Figure 3:**
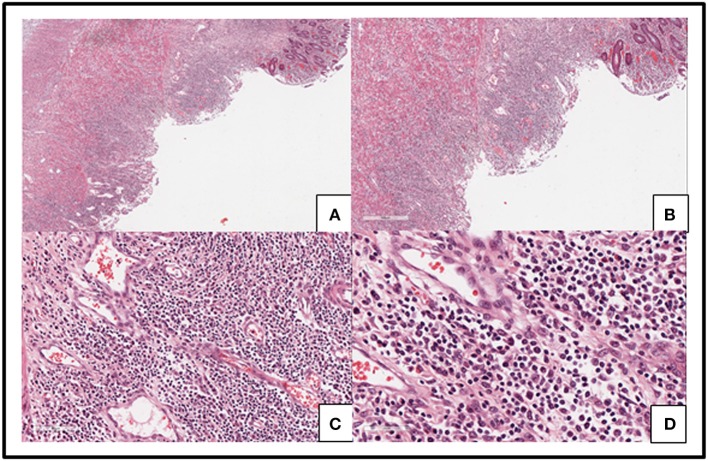
Slides from the resected colon showed ulceration of intestinal mucosa and intestinal interstitial edema **(A–D)** along with diffused infiltration of small-to medium-sized pleomorphic mild atypical lymphoid cells within mucosa and submucosa with a mixture of plasma cells and eosinophilic granulocyte and tissue cells **(C,D)**. Some lymphoid cells had big nucleus and obvious nucleoli **(D)**. Lymphoid cells were distributed in muscular layer and serosa **(A,B)**.

**Figure 4:**
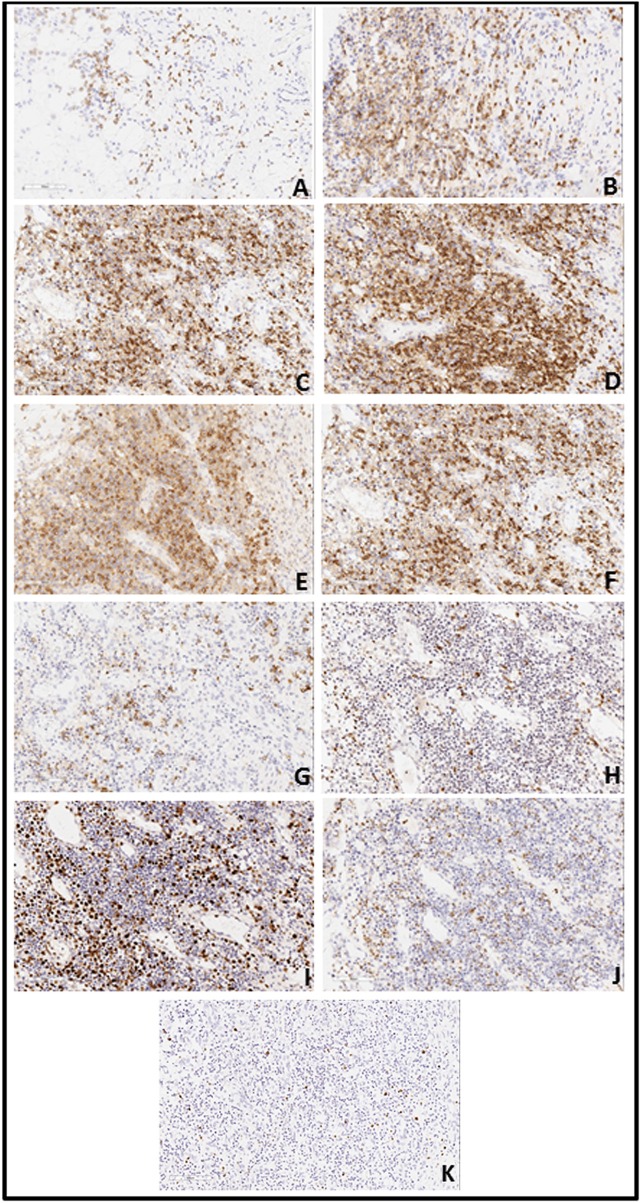
**(A)** Immunohistochemistry revealed a large number of atypical lymphoid cells expressing CD2 (**A**: 20X10), CD3 (**B**: 20X10), CD4(**C**: 20X10) and CD7 (**E**: 20X10). CD5 (**D**: 20X10)-positive cells were less than CD3-positive cells; CD8 (**F**: 20X10)-positive cells were less than CD4-positive cells and CD20 (**G**: 20X10)-positive cells presented a focal distribution. Granzyme B (**H**: 20X10)-positive cells were scattered. There was partial positivity for TIAI (**J**: 20X10), TCRGβ, and TCRγδ. Cells were negative for CD56 and Ki-67 (**I**: 20X10)-positivity was 40–50%. **(B)** EBV *in situ* hybridization for EBV encoded mi-RNA (EBER) demonstrated EBV-positive (50/HPF) atypical lymphoid cells (**K**: 20X10).

Capital Medical University-affiliated Beijing Friendship Hospital made a histological diagnosis of EBV T-cell LPD (II: Borderline) after histological examination of the resected tissue. Microscopic examination of resected colon slides revealed ulceration of intestinal mucosa and intestinal interstitial edema, which was accompanied by diffuse infiltration of small-to-medium-sized pleomorphic mild atypical lymphoid cells within mucosa and submucosa with a mixture of plasma cells and eosinophilic granulocyte and tissue cells. Some of the lymphoid cells had big nucleus and more obvious nucleoli. Lymphoid cells were observed to be distributed in muscular layer and serosa (Figure 3). We confirmed the diagnosis of EBV-associated T-cell LPD, based on the results from immunohistochemistry (IHC) and *in situ* hybridization of EBV-encoded miRNA (EBER). IHC revealed that the mild atypical lymphoid cells were positive for (Figure 4B) CD3, (Figure 4A) CD2, (Figure 4E) CD7, and (Figure 4C) CD4 expression. Further, a few atypical cells were also found positive for (Figure 4F) CD8, (Figure 4H) GranzymeB, (Figure 4J) TIAI, TCRGβ, and TCRγδ. The lymphoid cells were negative for CD56. (Figure 4I) Ki-67 positivity was 40–50%. *In situ* hybridization for (Figure 4K) EBER demonstrated EBV-positive atypical lymphoid cells of 50/HPF.

### Follow-up visits

**Figure 5:**
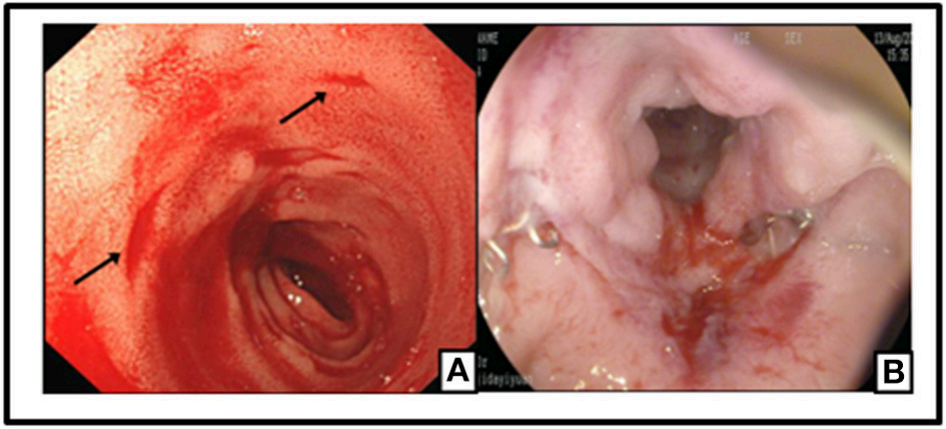
Multiple Aphthous bleeding ulcers scatted from the stoma to 40 cm away of small intestine (black arrow) **(A)**; Colonic post-operative anastomositis **(B)**.

**Figure 6:**
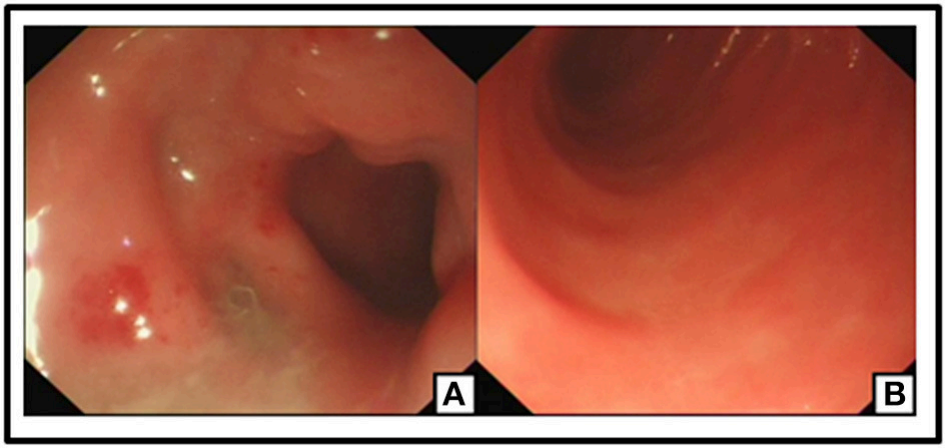
No ulceration or neoplasm was found in Endoscopy. Post-operation changes in small intestine **(A,B)**.

The patient was recommended to come back to hospital monthly for reexaminations. During these visits, she had signs of relapse every time, including new stoma ulcers and bloody stools. EB viral load test was done during her first follow-up visit, and EBV-DNA was found to be 2.55 × 10^6^ copies/ml for. A post-operative colonoscopy, performed at first relapse, showed multiple aphthous bleeding ulcers scattered from the stoma to about 40 cm away from small intestine, in addition to colonic post-operative anastomositis (Figure 5). Histological examination of biopsy samples confirmed the pathological diagnosis of EBV-T-cell LPD. Treatment with daily prednisolone 10 mg was initiated intravenously for a few days in the hospital. Oral prednisolone, 40 mg/day, was prescribed thereafter, after which it was tapered off slowly. The patient showed significant clinical improvement. Hematochezia was temporarily controlled until when the prednisolone was tapered off to 25 mg, and EBV-DNA decreased to 1.31 × 10^6^ copies/mL. Moreover, nothing remarkable was observed in colonoscopy this time (Figure 6). A bone marrow aspiration was strongly recommended but was not performed because of family refusal.

The last time patient came back to our department for reexamination was 4 months after her initial presentation. During this last visit, bone marrow aspiration was performed and found to be normal. However, liver function tests turned out to be abnormal (ALT 100 IU/L and AST 43 IU/L). EBV-DNA was 1 × 10^6^ copies/mL. The patient was referred to the Oncology Department of our Hospital for evaluation. She was asked to continue with the combination of prednisolone and anti-viral medication, until she presented with persistent fever and hematochezia, and died, 3 months later.

## Discussion

EBV is well-known to infect B-cells. It can infect T cells and NK cells as well. EBV-associated LPD constitutes a large group of disorders which are characterized by excessive lymphoid proliferation (2, 3, 6, 7). In recent years, there has been an increase in the number of adult patients developing systemic EBV+ T-cell LPD without immunodeficiency. Adult-onset cases are rare but the disease progresses rapidly. Only a small number of reports are available on the topic (3), which underlines the challenges for the clinicians providing care for such patients. Without doubt, further research on adulthood EBV T-LPD is required to illustrate differential diagnosis, especially the unique clinical presentations, all of which is needed for a better understanding of disease progression, and the best clinical management.

Although adulthood EBV T-LPD is systemic, it mostly involves liver, spleen and the heart (7, 8). The most common clinical symptoms reported are fever, liver dysfunction, hepatomegaly, splenomegaly, systemic lymphadenopathy and thrombocytopenia (2, 3, 9, 10). Only a few cases of adulthood EBV T-LPD, presenting with dominantly intestinal symptoms, have been reported around the world, according to literature review (7, 8, 11). Na et al. (7) reported two cases of immunocompetent patients with EBV-associated T-cell LPD of the small bowel and colon. These two patients were initially misdiagnosed for intestinal tuberculosis and Crohn's disease (CD). One of the patients was a previously healthy, 50 years old man presenting with general weakness, weight loss, loose stools, and fever. The other patient was a 49 years old woman presenting with recurrent hematochezia. Both patients had to undergo an emergency small bowel resection due to bowel perforation, and were given a definite diagnosis of EBV-associated T-cell LPD after histological examination of biopsy samples. Abdul-Ghafar et al. (8) reported one case of fulminant EBV-associated T-cell LPD in an immunocompetent middle-aged man, who presented with chronic diarrhea and gastrointestinal bleeding. Colonoscopy of this patient revealed multiple, variable sized and irregular shallow ulcerations from rectum to the ileocecal valve. He was initially misdiagnosed with infectious colitis and ulcerative colitis. However, a colonoscopy reexamination, along with histological examination of biopsy samples, helped reach a definite diagnosis of EBV-associated T-cell LPD. The patient was subjected to total colectomy because of uncontrolled hematochezia, and died with disseminated intravascular coagulation (DIC). Another case, reported by Sazuka et al. (11), was that of a 71 years old woman admitted with a 3-months history of severe diarrhea, weight loss and hypoalbuminemia. Capsule endoscopy performed on this patient revealed flattened villi throughout her small intestine. Further, double-balloon enteroscopy confirmed the diffusely atrophic small-intestinal villi and the clearly visible Peyer's patches. H&E (hematoxylin and eosin) staining of small-intestinal mucosa suggested celiac disease because of the presence of atrophic villous structures. The patient was put on a gluten-free diet for 1 month, but the symptoms did not alleviate. Further tests, such as, EBV serology test, EBV-DNA, southern blot analysis of EBV-infected cells, fluorescence activated cell sorting (FACS) and EBV-encoded RNA *in situ* hybridization helped make the final diagnosis of EBV-associated T-cell LPD. Despite sequential treatment with cyclosporine and CHOP, the patient died 5 months later. Lastly, Zheng et al. (12) reported one case of an immunocompetent patient with EBV-associated T-cell LPD of the colon. This 26 years old male patient had been initially misdiagnosed with ulcerative colitis (UC). The patient presented with diarrhea and hematochezia and reported intermittent high fever for more than 3 months. The patient ended up being diagnosed with EBV+ T-cell LPD (Grade II: borderline lesions), based on the findings from morphological determinations, IHC, ISH for EBER and TCR gene rearrangement. Right hemicolectomy was performed on this patient due to active hemorrhage of colonic ulcers, but the patient died 2 months after the EBV+ T-cell LPD diagnosis.

There is limited knowledge on intestinal lesions in adult-onset systemic EBV T-LPD. Some patients have been reported to present with mild symptoms like chronic diarrhea, while others had a fulminant clinical course with complications like intestine perforation and severe gastrointestinal bleeding resulting in the death of the patient within a time period of few days to few months, post- diagnosis. It is apparent that early diagnosis and appropriate treatment is critical. Multiple ulcerations with surrounding edematous and erythematous mucosa can often be seen during colonoscopy. Both, small bowel and colon could be involved, and there is reported variation in shape, depth and size of the ulceration. Histological analysis of the ulcerated mucosa can reveal the extent of proliferation of the various lymphocytes, in addition to identifying cells as normal or atypical. As discussed above, this disease can be easily misdiagnosed as intestinal tuberculosis (ITB) and inflammatory bowel diseases (IBD) because of its atypical colonoscopy manifestations and clinical features, especially when the biopsy tissue is inadequate and a few atypical lymphoid cells could be found in the specimens. Symptoms like fever, lymphadenopathy, diarrhea and anemia can often be seen in TB and IBD patients which makes the differential diagnosis more difficult and challenging.

Despite the resembling colonoscopy lesions, there are some specific clinical manifestations, endoscopic and pathological findings of TB or IBD, respectively. Colonoscopy of TB usually presents with transverse ulcers which are shallow and irregular, with involvement of ileocecal area. Further, caseous granulomas and acid-fast stain positivity are typical pathological findings. A reliable diagnosis of ITB can be based on a combination of symptoms and tests, such as night sweats, concomitant pulmonary tuberculosis, positive tuberculin skin test, positive antibody to tuberculosis and abdominal lymphadenopathy and ascites. Although the discrete ulcers in the case presented here mimicked lesions found in CD, her endoscopic features were distinct from those of CD ulcers, primarily because longitudinal ulcers, the ones with cobblestone appearance or longitudinally arranged aphthous ulcers, were not visible. Non-caseous granulomas are specific to CD. Hematochezia, intestinal obstruction, fistula, etc. are common complications in CD patients (13). A positive diagnosis for UC is made based on presentation of symptoms and the endoscopic evidence of continuous colonic mucosal inflammation with fresh blood and suppuration on it, which almost always begins in the rectum and extends proximally. Biopsy specimens are evaluated as a means to further confirm the diagnosis and should not be a surrogate for diagnosis themselves. These specimens help establish chronic active colitis. Histologically speaking, the disease is limited to mucosal layers, with varying degrees of infiltrates from lymphocytes, plasma cells, and granulocytes. Other histologic findings include distortion of the crypt architecture with shortening and disarray of the crypts, crypt atrophy, crypt abscesses, and crypt branching (14). Sometimes, the diagnosis of IBD can be made easily, but a classification diagnosis of IBD is difficult to confirm because the two main types, ulcerative colitis (UC) and Crohn's disease (CD), share similar symptoms and are indistinguishable. Indeterminate colitis (IC) and inflammatory bowel disease unclassified (IBDU) are the disease conditions often diagnosed in such difficult to diagnose patients (15).

Intestinal EBV-T-LPD limited within small intestine is extremely rare and there is only one reported case in the literature (11). The change within the intestine is not ulcerative mucosa but diffusely atrophic small-intestinal villi and clearly visible Peyer's patches, as revealed by double-bloon endoscopy, tentatively suggesting celiac disease. Celiac disease happens to be one of the most common causes of chronic malabsorption. The failure in absorption of adequate calories results in loss of weight, and such malabsorption leads to abdominal pain and bloating (16). Thus, a gluten-free diet treatment can help differentiate celiac disease from small intestinal EBV-T-LPD, as only celiac disease patients will show great symptom alleviation.

Histopathology tests play a critical role in making a definite diagnosis for EBV-T+LPD. They help determine the various degrees of T-lymphocytes proliferation, in addition to finding atypical cells. This is important because no atypical lymphocytes exist in IBD patients (17). Ohshima et al. (9) reported a study that evaluated 108 cases of EBV-T/NK-LPD. All the cases were reported to be positive for cytotoxic molecules which indicated their cytotoxic T cells origin. The IHC analysis of our patient revealed that a few atypical cells were positive for CD8, GranzymeB, TIAI, TCRGβ, and TCRγδ, which is similar to the findings described in Ohshima et al. study. It is now widely believed that correct diagnosis of EBV-T+LPD is reached through the integration of histopathology, IHC, and EBER and clonality analysis in the context of clinical history and laboratory data of EBV infection (including antibody profile and EBV DNA load in the serum) (6).

Currently, there is lack of standard treatment protocol for systemic EBV-T-LPD patients. Various therapies have been tested, which include, antiviral, corticosteroids, chemotherapy and immunomodulatory drugs. However, the success has been very limited with minimal remission. Such therapeutic approaches resulted in complete remission in only rare cases and, presently, HSCT is the only curative therapy (2, 3, 6, 18), even though HSCT is known to be associated with substantial risk of transplantation-related complications. It is often difficult for patients to consent for HSCT especially when the symptoms are not very severe (3). The preclinical testing of two candidate drugs for this disease has recently been reported with some encouraging results. One of those new drug is Bortezomib, which is a known 26S proteasome inhibitor. It can induce apoptosis and the expression of EBV lytic-cycle genes *BZLF1* and *gp350/220* in various human T-cell lymphoma cell lines (19). The other new drug is Valproic acid, a widely used anti-epileptic drug and a known inhibitor of histone deacetylases (HDAC). Iwata et al. (20) reported induction of apoptosis by valproic acid in human EBV-infected T cells. To summarize, adult systematic EBV-T-LPD, with an onset of intestinal presentations, mostly has a fulminant clinical course and progresses rapidly. Owing to severe complications, patients do not have much choices, and have to undergo surgery. Early detection of this disease, along with a correct diagnosis, can afford the much needed time for a conservative and effective treatment. While some progress has been recently reported, further studies to identify novel drugs and treatment strategies are urgently needed.

## Author contributions

YuL was the attending physician of this patient and the director of the whole writing process. MW and WC participated in all data collection and processing. XD was responsible for reading and interpreting the pathological images. TT performed the colonoscopy examination. YW, YaL, and XM were the major contributors in organizing records and drafting the manuscript. All authors proofread and approved the manuscript.

### Conflict of interest statement

The authors declare that the research was conducted in the absence of any commercial or financial relationships that could be construed as a potential conflict of interest.
